# A simple custom appliance against droplet and aerosol transmission of COVID-19 during advanced airway management

**DOI:** 10.1186/s13054-020-02985-5

**Published:** 2020-06-08

**Authors:** Meng Chi, Changming Lou, Xiuli Zhao, Xin Sui, Fei Han

**Affiliations:** 1 Department of Anesthesiology, The Third Affiliated Hospital, Harbin Medical University, 150 Haping Road, Harbin, 150081 Heilongjiang China; 2 Department of Anesthesiology, The Pingle Orthopedic Hospital of Shenzhen, Shenzhen, 518010 Guandong China; 3 Department of Anesthesiology, The Fifth Hospital of Harbin, Harbin, 150040 Heilongjiang China

**Keywords:** COVID-19, Protective measures, Custom appliance, Aerosol, Droplet

Health care workers are exposed to high-risk environments when patients infected with COVID-19 require advanced airway management. The virus tends to be transmitted in the air by droplets and aerosols during these procedures [1, 2]. It was demonstrated that a mask over a patient’s nose and mouth to prevent airborne transmission was effective in protecting health care workers [3]. However, patients cannot wear masks during positive pressure ventilation and endotracheal intubation, exposing health care workers to high-risk environments, even if effective protective measures were taken.

One study showed that when a human-patient simulator simulated a cough in the supine position, the aerosol spread in the sagittal plane by approximately 86 cm [4]. The propagation distance reached 26.7 cm during mask positive pressure ventilation. The spread range of a cough reached 46 cm after endotracheal intubation. During endotracheal intubation, the operator is directly exposed to the front of the patient’s respiratory tract. A large amount of aerosols is spread to the operator’s face, hands, and surrounding equipment, seriously increasing the operator’s risk of infection. Therefore, the establishment of a barrier between the patient and the operator can decrease the diffusion area of the patient’s exhaled gas, thus greatly reducing the concentration of droplets and aerosols in the surrounding environment and the risk of infection of the operator.

We use the PVC membrane as a barrier that is easy to fabricate during advanced airway management (Fig. 1a–d). It is used to block the spread of droplets and aerosols, and operators can view patients easily because the membrane is waterproof and transparent. The PVC membrane should be sufficiently large (> 100 cm × 100 cm is recommended) to cover the head of the patient and have a hole (sealed when necessary) in the center for the connection between the face mask and the circuit during oxygen inhalation and positive pressure ventilation. Before advanced airway management, the PVC membrane with an assembled face mask under the membrane is placed over the patient’s mouth and nose for the induction of intubation (Fig. 1a). The artificial nose and the circuit of the ventilator are on the operator’s side. There is the other hole (sealed when necessary) of the PVC membrane for laryngoscopy insertion and intubation (Fig. 1b). Sedatives and muscle relaxants are given to the patient to reduce the cough and choking responses during intubation. The appliance covers the head and face of the patient throughout the intubation, the mechanical ventilation (Fig. 1c), and the extubation periods. The mask is reapplied to the patient for oxygen inhalation after extubation (Fig. 1d).

**Fig. 1:**
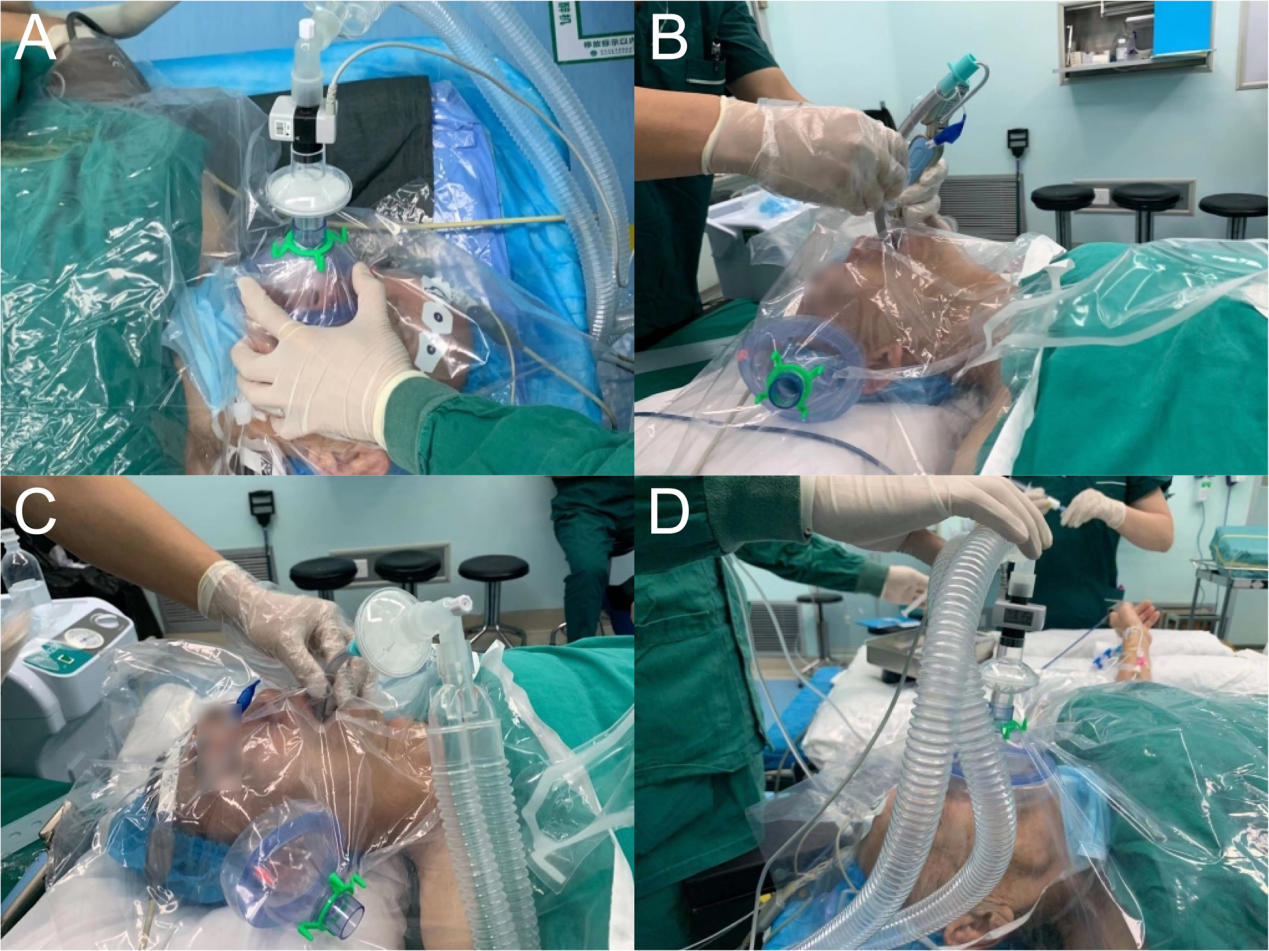
The process of advanced airway management from intubation to extubation. The photographs are demo on a non-COVID-19 patient. **a** Positive pressure ventilation before intubation. **b** Intubation. **c** Mechanical ventilation. **d** Oxygen inhalation after extubation

The custom appliance theoretically reduces the spread of droplets and aerosols originating from patients, reducing the risk of infectious disease transmission. First, the barrier acts as a mask and an isolation ward, reducing the spread of droplets and aerosols in the air, and it simply isolates the patient, reducing the potential for air transmission. Second, the barrier covers the patient, which greatly reduces the risk of contact between the operator and the patient and blocks the transmission route of the virus by contact. Finally, this device is inexpensive and easy to apply. It does not require any additional training.

In view of the high infection and transmission rate of COVID-19, it is equally important to control the source of infection and enhance the protective measures of health care workers. Our appliance reduces the spread of droplets and aerosols from patients, blocking the airborne transmission route of the virus to a large extent and providing a new layer of protection for health care workers during advanced airway management.

## Data Availability

Not applicable.
